# Inflammatory Biomarkers and Their Associations with Arrhythmic Burden Following SGLT2-I Treatment in Chronic Heart Failure—A Subanalysis of the ERASe Trial

**DOI:** 10.3390/jcm15124681

**Published:** 2026-06-17

**Authors:** Martin Benedikt, Markus Herrmann, Faisal Aziz, Norbert J. Tripolt, Peter Pferschy, Martin Manninger, Markus Wallner, Raffaela Planka, Andreas Zirlik, Harald Sourij, Abderrahim Oulhaj, Markus Stühlinger, Daniel Scherr, Dirk von Lewinski

**Affiliations:** 1 Department of Internal Medicine, Division of Cardiology, Medical University of Graz, 8010 Graz, Austria; martin.benedikt@medunigraz.at (M.B.); martin.manninger@medunigraz.at (M.M.); markus.wallner@medunigraz.at (M.W.); raffaela.planka@stud.medunigraz.at (R.P.); andreas.zirlik@medunigraz.at (A.Z.); daniel.scherr@medunigraz.at (D.S.); 2 Clinical Institute of Medical and Chemical Laboratory Diagnostics, Medical University of Graz, 8010 Graz, Austria; markus.herrmann@medunigraz.at; 3 Department of Internal Medicine, Division of Endocrinology and Diabetology, Medical University of Graz, 8010 Graz, Austria; faisal.aziz@medunigraz.at (F.A.); norbert.tripolt@medunigraz.at (N.J.T.); peter.pferschy@medunigraz.at (P.P.); ha.sourij@medunigraz.at (H.S.); 4 Cardiometabolic Trials Unit, Medical University of Graz, 8010 Graz, Austria; 5 Department of Public Health and Epidemiology, College of Medicine and Health Sciences, Khalifa University of Sciences and Technology, Abu Dhabi 127788, United Arab Emirates; abderrahim.oulhaj@ku.ac.ae; 6 Biotechnology Centre, Khalifa University of Sciences and Technology, Abu Dhabi 127788, United Arab Emirates; 7 Department of Internal Medicine, Division of Cardiology, Medical University of Innsbruck, 6020 Innsbruck, Austria; markus.stuehlinger@tirol-kliniken.at

**Keywords:** Ertugliflozin, arrhythmia, inflammation, chronic heart failure, high-sensitive C-reactive protein

## Abstract

**Background**: Sodium glucose-linked transport 2 inhibitors (SGLT2-Is) are well known to exert beneficial effects in chronic heart failure (CHF) independent of left ventricular ejection fraction (LVEF). As inflammation plays a key role in cardiac diseases, data on the association of inflammatory biomarkers and ventricular arrhythmic (VA) burden in SGLT2-I-treated patients is lacking. **Methods:** This pre-defined subanalysis investigated changes in pre-specified inflammatory biomarkers from baseline to week 52 in response to 5 mg Ertugliflozin compared to placebo and their associations to the incidence of VA burden. **Results**: A total of 36 patients (18 versus 18) with available biobank samples were included in the analysis. At week 52, leukocyte and neutrophil counts, as well as high-sensitive C-reactive protein (hsCRP) and interleukin-6 (IL-6), were numerically higher in the Ertugliflozin group. In contrast, neutrophil-to-lymphocyte ratio (NLR) and platelet-to-lymphocyte ratio (PLR) were lower in the Ertugliflozin group, although these differences did not reach statistical significance. Notably, lymphocyte counts were significantly higher in Ertugliflozin showing a mean difference of 19.0 ± 10.78% (*p* = 0.028). Further, a significantly higher incidence of VA burden was observed among Ertugliflozin-treated patients with elevated hsCRP levels (incidence rate ratio [IRR] 3.58; 95% Confidence interval [CI], 1.12–11.40, *p* = 0.031). **Conclusions**: In patients with CHF, Ertugliflozin treatment was associated with a higher incidence of VA burden in those with elevated hsCRP levels. This may suggest a potential higher risk for VA in SGLT2-I-treated patients in the setting of heightened inflammatory activity. However, this finding is based on a single interaction analysis in a small sample size, and the results should therefore be considered exploratory and hypothesis-generating, and must be interpreted cautiously.

## 1. Introduction

Sodium glucose-linked transporter 2 inhibitors (SGLT2-Is) were identified to exert beneficial effects on cardiovascular outcome, including cardiovascular death and hospitalisation for heart failure (HFH), in patients with chronic heart failure with reduced ejection fraction (HFrEF), independent of diabetic status [1,2,3]. Similar findings have been reported forSGLT2-Is in patients with chronic heart failure with mildly reduced and preserved ejection fraction (HFmrEF and HFpEF) [4,5] and hence, received a Class IA recommendation for the treatment of chronic heart failure, irrespective of LVEF [6,7]. Recently published data also revealed significant improvement in structural and functional cardiac markers, N-terminal pro-brain natriuretic peptide (NTproBNP) levels, as well as cardiometabolic outcomes in SGLT2-I-treated patients following acute myocardial infarction (AMI) [8,9]. However, large clinical outcome trials have failed to demonstrate benefits on long-term cardiovascular outcome [8,10].

Inflammation plays a crucial role in the development and progression of cardiovascular diseases, particularly in atherosclerosis and AMI [11,12]. Numerous cytokines, such as interleukin-6 (IL-6), and acute phase reactants, including high-sensitive C-reactive protein (hsCRP), are centrally involved in this complex inflammatory process [13,14,15]. In large clinical outcome trials, elevated levels of serum hsCRP were associated with the worsening of cardiovascular outcome by significantly increasing the risk for major adverse cardiovascular events (MACE), cardiovascular death, as well as all-cause mortality [16,17,18] following AMI. Similarly, elevated levels of serum IL-6 and IL-1 have been shown to enhance ventricular dysfunction by promoting adverse cardiac remodelling after AMI [19,20,21]. Targeting IL-6 and IL-1 pathways has been associated with a decrease in serum hsCRP levels and significant improvement of cardiovascular outcome post-AMI, suggesting promising therapeutic approach in this area [22,23]. In the RESCUE trial, the IL-6-modulating agent ziltivekimab significantly reduced serum hsCRP levels compared with placebo in patients at high atherosclerotic risk going in line with a reduced risk for heart failure [24]. Further, the IL-1 receptor antagonist Anakinra was found to reduce inflammation and improve rehospitalisation rates for heart failure in patients with HFrEF and recent decompensation [25].

Although several trials have demonstrated beneficial effects of anti-inflammatory therapies on cardiovascular outcome, data on the complex relationship between inflammation and arrythmias in CHF patients remain limited, despite inflammation playing a crucial role in both the initiation and maintenance of arrhythmias [26]. Experimental studies identified that inflammatory cytokines, including IL-1ß, tumour necrosis factor alpha (TNFα) and IL-6, can modulate cardiac ion channels and thereby increase the risk for VA [27]. Proposed mechanisms include alterations in transient outward potassium channels [28], rapid delayed-rectifier potassium channels [29], L-type calcium channels [30], Ca2+-ATPase activity, and sarcoplasmic reticulum calcium leak [28,31].

Within the ERASe (Ertugliflozin to reduce arrhythmic burden in patients with implanted cardioverter defibrillator [ICD] ± cardiac resynchronisation therapy [CRT]) trial, we provided the first evidence of beneficial effects of the SGLT2-I Ertugliflozin on the incidence of VA burden in patients with chronic heart failure and reduced LVEF, who had an implanted ICD or CRT-D [32]. However, data concerning the impact of inflammatory biomarkers on VA burden in SGLT2-I-treated patients remain scarce. Given that SGLT2-Is may influence both inflammatory pathways as well as arrhythmic burden, a potential association would be reasonable and warrants further investigation.

This post hoc analysis of the ERASe trial aimed to investigate inflammatory biomarkers, their changes following SGLT2 treatment in patients with CHF, and their association with the incidence of VA burden.

## 2. Material and Methods

### 2.1. The ERASe Trial

The “Ertugliflozin to Reduce arrhythmic Burden in patients with ICDs ± CRTs” (ERASe) trial was an investigator-initiated, multicentre, randomised, double-blind and placebo-controlled phase 3b trial investigating the effects of 5 mg Ertugliflozin on the incidence of VA burden in patients with HFrEF or HFmrEF and ICD ± CRT-D over 52 weeks, compared to placebo (see CONSORT checklist at the Supplementary Files). The trial was approved by the relevant regulatory authorities, by the Ethics Committee of the Medical University of Graz, Austria, (EK 32-492 ex 19/20) EudraCT 2020-002581-14 and was registered on ClinicalTrials.gov (NCT04600921). The ERASe trial was conducted in full conformity with the 1964 declaration of Helsinki and all subsequent revisions, as well as in accordance with the guidelines laid down by the International Conference on Harmonisation for Good Clinical Practice (ICH GCP E6 guidelines) [33].

### 2.2. Study Cohort

Within ERASe, we prospectively enrolled 46 patients at eight Austrian sites between 24 June 2021 (first patient first visit) and 23 June 2023 (last patient last visit); however, the trial was terminated early due to changes in guidelines, with SGLT2-Is receiving a Class IA recommendation for the treatment of CHF irrespective of LVEF. This post hoc analysis included all patients with available biobank samples from the baseline visit and week 52. Patients aged between 18 and 80 years with a LVEF < 50% despite optimal chronic heart failure treatment, an estimated glomerular filtration rate (eGFR) > 30 mL/min/1.73 m^2^ and ICD ± CRT-D for more than three months, who suffered from at least 10 episodes of ventricular tachycardia (including non-sustained ventricular tachycardia [nsVT] or sustained ventricular tachycardia [sVT]) within the last 12 months, with or without ICD therapy, were eligible for inclusion in the ERASe trial. Further, the enrolled patients need to fulfil at least one of the following additional criteria: N-terminal pro b-type natriuretic peptide (NTproBNP) > 500 pg/mL, LV-EF < 35%, hospitalisation for heart failure within the last 12 months, >100 episodes of nsVT within the last 12 months, or more than one episode of sVT within the last 12 months. Exclusion criteria included hemodynamic instability with a systemic arterial pressure < 100/60 mmHg requiring inotropic support (catecholamines, calcium sensitisers, and phosphodiesterase inhibitors) or ongoing ventricular tachycardia (VT), allergy or intolerance to SGLT2-Is, ongoing SGLT2-I treatment, other forms of type-2 diabetes mellitus (T2DM), a history of ketoacidosis, planned VT ablation or device explantation, as well as history of at least one hypoglycaemic event within the least six months under treatment with insulin or sulfonylurea [33].

VA burden was defined as the total number of sVT/VF episodes recorded during device interrogation at baseline and follow-up. Sustained ventricular tachycardia was defined as a VT lasting > 30 s, in accordance with current guidelines [34].

### 2.3. Study Design

After enrolment and submission of written informant consent (IC), patients were randomised to receive either 5 mg Ertugliflozin or a placebo orally once daily. Visits were carried out with two on-site visits (baseline and after 52 weeks), including device interrogation and blood samples, as well as one telephone visit 4 weeks after the last follow-up. Biobank samples were collected from each patient across eight Austrian sites at baseline and after 52 weeks and were analysed in local laboratories at the respective trial visits. For this post hoc analysis, frozen blood samples were stored at the Medical University of Graz for proper analysis [33].

### 2.4. Study Variables

In this post hoc analysis of the ERASe trial, outcome variables included serum levels of inflammatory biomarkers, such as leukocyte count, neutrophil count, lymphocyte count, hsCRP, IL-6, neutrophil-to-lymphocyte ratio (NLR) and platelet-to-lymphocyte ratio (PLR), defined as mean changes from baseline to week 52. Frozen blood samples were collected at baseline as well as week 52 in all participating centres, centrally analysed and measured on the automated platform Cobas 8000, c-modul 702 (Roche Diagnostics, Mannheim, Germany). The applied methods included the Elecsys IL-6 sandwich assay and Tina-quant C-Reactive Protein IV particle enhanced immunoturbidimetric assay technology (Roche Diagnostics, Mannheim, Germany). Leukocytes, including the respective subfractions, were counted on a Sysmex CN 6000 analyser (Sysmex Corporation, Austria). All analyses were performed at the central laboratory of the Medical University of Graz.

### 2.5. Statistical Analysis

A complete case analysis of inflammatory biomarkers in all enrolled patients participating in the ERASe trial, with available frozen blood samples from baseline visit and at week 52, was included in this post hoc analysis. All statistical analyses were conducted in the Stata software version 18.0.

Baseline parameters were calculated using descriptive analysis and presented as mean ± standard deviation (SD) or median ± interquartile range (IQR) for continuous variables and compared with the placebo group using the Wilcoxon rank-sum test. Categorical variables were summarised as frequences or percentages (%) and compared with the control group using Fisher’s Exact test or Chi-Square test. Baseline characteristics were reported with 95% confidence interval (CI), and a *p*-value < 0.05 was considered statistically significant.

Linear regression was performed to compare treatment effects with inflammatory markers adjusted for baseline values. Results were reported as mean ± standard error of mean (SEM) with a confidence interval (CI) of 95% as well as a *p*-value < 0.05 for statistical significance.

Negative binominal regression analysis was performed to assess the association of change in biomarkers from baseline to 52 weeks and the incidence of VA burden. Treatment–biomarker interaction and baseline ventricular episodes were added as covariates in the model. The log time to ventricular burden was added as an offset variable in the model. The model was not further adjusted for any covariates. Results of binomial regression were reported as an incidence rate ratio (IRR) with a confidence interval of 95% and a level of significance *p* < 0.05.

All tests and analyses were conducted in the Stata software version 19.0. 

## 3. Results

### 3.1. Trial Population

Within the published ESC guidelines in 2021 for heart failure, SGLT2-Is received a Class IA recommendation for the treatment of chronic heart failure with reduced ejection fraction, followed by the focused ESC Update 2023, which extended the Class IA recommendation to chronic heart failure with mildly and preserved ejection fraction [6,7]. Consequently, these changes in guidelines led to an early termination of the ERASe trial.

**Figure 1:**
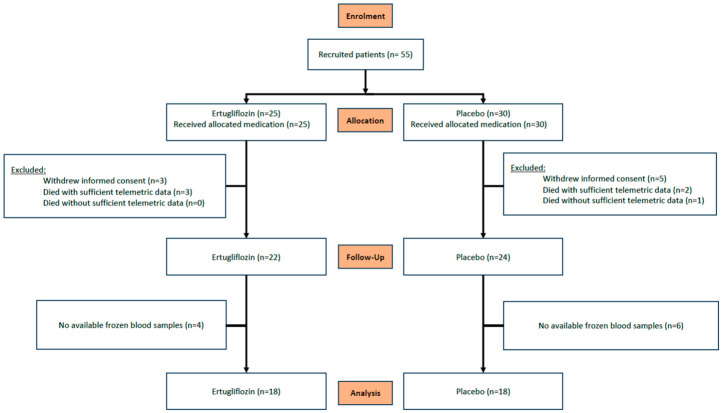
Flowchart of the post hoc analysis study cohort.

Within the entire ERASe cohort, 55 patients were successfully enrolled to receive either 5 mg Ertugliflozin or a placebo; however, eight patients withdrew the informed consent, and one patient died before trial termination [32]. Among the remaining 46 patients (22 in the Ertugliflozin group and 24 in the placebo group), 36 patients (18 Ertugliflozin and 18 placebo) had available biobank samples from both baseline and follow-up visit and were included in the inflammatory subanalysis (Figure 1).

Most baseline characteristics were well balanced between the two treatment groups. The median age in the overall study population was 64 years (IQR 58–75), with a median body mass index (BMI) of 27 kg/m^2^ (IQR 25–32), and 92% of all participants were male. Median systolic blood pressure was 137 mmHg (IQR 127–147), median diastolic blood pressure was 84 mmHg (IQR 75–97), and median heart rate was 67 beats per minute (bpm) (IQR 59–77). Cardiovascular risk factors were similar between both groups, with arterial hypertension present in 25 (69%) patients, diabetes in 4 (11%) patients, and a history of smoking in 24 (67%) patients. Prior to randomisation, 84% of the patients received an angiotensin-converting enzyme inhibitor (ACE-I), angiotensin receptor blocker (ARB) or angiotensin receptor neprilysin inhibitor (ARNI); 56% received a mineralocorticoid receptor antagonist (MRA), and 83% received a betablocker (BB). No patient in the trial received class I anti-arrhythmic drugs; 19% of the participants were treated with amiodarone. Heart failure markers were also equally distributed in both treatment groups, with a mean LVEF of 34.75% (IQR 28–43), and a median NTproBNP level of 551 ng/L (241–2562) (see Table S1: Baseline characteristic of the post-hoc analysis).

### 3.2. Changes in Inflammatory Markers

**Figure 2:**
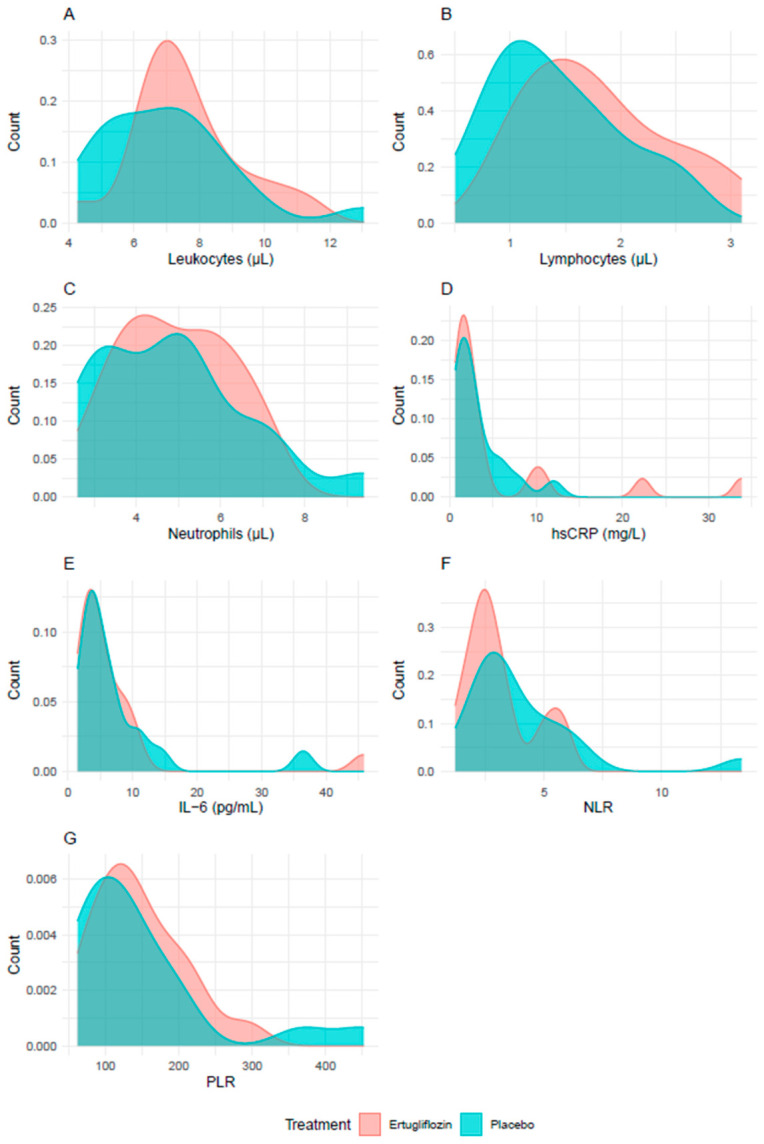
Inflammatory markers between treatment arms at follow-up. (**A**) = Leucocytes; (**B**) = Lymphcytes; (**C**) = Neutrophils; (**D**) = hsCRP; (**E**) = IL-6; (**F**) = NLR; (**G**) = PLR; Abbreviations: hsCRP = high-sensitive C-reactive protein; IL-6 = interleukin-6 = NLR, neutrophil–lymphocyte ratio; PLR = platelet–lymphocyte ratio.

At week 52, absolute leukocyte and neutrophil counts were found to be numerically higher in the Ertugliflozin group, without significant differences between the groups, with a mean relative difference of 7.0 ± 6.3% for leukocytes (*p* = 0.223) and a mean difference of 6.0 ± 8.3% for neutrophils (*p* = 0.460). In contrast, lymphocyte counts were found to be significantly higher in the Ertugliflozin group, with a mean difference of 19.0 ± 10.78% (*p* = 0.028) at week 52. Inflammatory biomarkers were numerically higher in the SGLT2-I group, revealing a mean difference of 21.0 ± 45.9% for hsCRP and 10.0 ± 25.0% for IL-6, respectively, but without a significant difference at week 52 compared to placebo (*p* = 0.558 and *p* = 0.631). NLR and PLR were numerically lower in the Ertugliflozin group, with a mean difference of −13.0 ± 8.9% for the NLR (*p* = 0.256) and a mean difference of −11.0 ± 7.68% for the PLR (*p* = 0.247) (Table 1 and Figure 2).

### 3.3. Correlation Analysis

Negative binomial regression analysis of associations between VA burden episodes at follow-up and changes in inflammatory biomarkers revealed an incidence rate ratio (IRR) of 1.33 (95% CI, 0.86–2.13, *p* = 0.118) for leukocyte counts, 2.12 (95% CI, 0.35–13.5, *p* = 0.339) for lymphocyte counts, 1.43 (95% CI, 0.88–2.50, *p* = 0.056) for neutrophil counts, 1.16 (95% CI, 0.52–2.33, *p* = 0.623) for hsCRP, 1.39 (95% CI, 0.28–6.81, *p* = 0.608) for IL-6, 0.98 (95% CI, 0.66–1.70, *p* = 0.882) for NLR, and 1.00 (95% CI, 0.98–1.03, *p* = 0.487) for PLR. Interaction analysis demonstrated a significantly higher incidence of VA burden in Ertugliflozin-treated patients only with elevated hsCRP levels (IRR 3.58; 95% CI, 1.12–11.40, *p* = 0.031); however, no further significant interaction was observed between the incidence of VA and other inflammatory metrices (Table 2).

## 4. Discussion

The ERASe trial was the first clinical trial worldwide showing beneficial effects of the SGLT2-I Ertugliflozin on the incidence of VA burden in patients suffering from chronic heart failure and reduced ejection fraction, who had an ICD ± CRT, by significantly reducing the total number of sVTs/VF and nsVTs compared to placebo [32]. Due to the recent changes in chronic heart failure guidelines in 2023 [6], the trial was terminated earlier after enrolment of 11% (*n* = 46) of the planned patients; however, only 36 were included in this post hoc analysis. As guidelines have changed for SGLT2-Is, receiving a Class IA recommendation of chronic heart failure irrespective of LVEF, it is unlikely that any further randomised, clinical placebo-controlled trials in this area will be performed.

Ventricular arrythmias are common in patients with chronic heart failure and reduced ejection fraction, and first data about the incidence of arrhythmias arrived from the DAPA-HF trial demonstrating a 21% reduction in the composite of serious VA, resuscitated cardiac arrest, or sudden death [35], while smaller studies also reported a reduced VA burden in SGLT2-I-treated patients [36,37]. As inflammation appears to be a key driver in the development of CHF, especially following acute myocardial infarction, SGLT2-Is were found to have pleiotropic effects, including anti-inflammatory, anti-fibrotic, energetics-modifying, as well as metabolic effects affecting the myocardium, which positively exert cardiac remodelling and might reduce VA burden [38,39,40,41,42]. Although inflammation is a key driver for cardiovascular outcome, little is known about the relationship between inflammation and VA burden, which affects both duration as well as onset of arrhythmias [26].

The main pro-arrhythmogenic mechanism is probably driven by the direct cardiac effects of inflammatory cytokines. These include membrane iodine channel modulation followed by prolongation of the ventricular action potential duration, modulation of intracellular calcium haemostasis with spontaneous diastolic calcium release from the sarcoplasmic reticulum, connexin changes followed by dysfunction of the gap junctions, as well as the induction of myocardial fibrosis [43].

Although several anti-arhythmic properties of SGLT2-Is have been reported, the main mechanism still remains unclear. Proposed targeting pathways include modulation of sodium and calcium haemostasis through inhibition of the Na+/H+ exchanger 1 (NHE1) and late Na+ (late I_Na_) [44,45], enhancements of voltage-gated potassium (K+) currents [46], as well as attenuation of myocardial fibrosis [45,47]. Additionally, SGLT2-Is increase ketone bodies availability, particularly ß-hydroxybutyrate, which may improve myocardial energy supply and exert anti-inflammatory effects through suppression of proinflammatory cytokines and inhibition of the NLRP3-inflammosome, both contributing to improvement of cardiac remodelling [48,49,50,51,52].

Previous studies reported a pro-arrhythmic role of inflammatory markers in patients with structural heart disease. The biomarkers hsCRP, NLR and neutrophil counts were found to be positively associated with the incidence of malignant VA in patients with structural heart disease [53,54]. Further, IL-6 was also identified as a predictor for spontaneous ventricular arrhythmic events in ICD patients with CAD [43]. In our subanalysis numerically higher levels of inflammatory markers were found for Ertugliflozin without a significant difference compared to the placebo, and thus, Ertugliflozin does not confer anti-inflammatory benefits in CHF patents with high VA burden; however, this finding might be in line with the small sample size of the groups (Figure 2).

SGLT2-Is have been shown to attenuate Th17/Treg imbalance in diabetic mouse models, primarily through a decrease in proinflammatory Th17 cells and an increase in T-regulatory cells (Treg), suggesting potential anti-inflammatory effects [55,56]. In our study, lymphocyte counts were significantly higher in the SGLT2-I group at week 52, which may similarly indicate anti-inflammatory and potentially cardioprotective effects in our patients; however, specific T-cell subpopulations were not assessed in this trial. Furthermore, platelet count as well as PLR have previously been identified as predictors of worsening of LVEF and HF outcome, whereas studies regarding their association with ventricular arrythmias remain limited [57]. In our analysis, significant higher baseline platelet counts were observed in the Ertugliflozin group (*p* = 0.001); however, PLR showed no significant differences between groups or in interaction analysis. Although these findings reached statistical significance, all other inflammatory markers showed no consistent difference, indicating that the results may be in the context of the small sample size. Accordingly, the findings should be considered exploratory and hypothesis generating.

Interaction analysis demonstrated a higher VA burden in Ertugliflozin-treated patients with elevated hsCRP levels, whereas no consistent association was found for other inflammatory metrices. Although SGLT2-Is revealed cardioprotective effects, including attenuating inflammatory response in patients with CHF [51] as well as a significant reduction in VA burden in the main trial [33], this subanalysis may suggest a potential increased risk of VA in Ertugliflozin-treated patients with CHF with heightened inflammatory activity. This may be explained by the fact of higher risk for major complications in SGLT2-I-treated patients suffering from acute critical illness or severe infections, potentially mediated by increased sympathetic tone, and highlights the importance of temporary discontinuation of SGLT2-Is in such patients [58]. However, this hypothesis is not directly supported by our data and is based only on a single significant interaction with wide confidence interval, while no further significant relations were observed for other inflammatory markers. This suggests that the finding may be incidental in the context of the small sample size, and the results should be interpreted with caution. Further, this subanalysis is underpowered for multiple-association testing, and findings should be considered exploratory as well as hypothesis generating.

Despite these mechanisms, the main anti-arrhythmic effect of SGLT2-Is still remains unclear, but attenuation of inflammatory response appears to play a major role in cardioprotection; however, further research in this area is required.

## 5. Strengths and Study Limitations

ERASe was prematurely terminated due to changes in guidelines for SGLT2-Is receiving a Class IA recommendation for treatment of chronic heart failure irrespective of LVEF and diabetic status [31] and thus, was underpowered for accurately assessing the primary endpoint.

In the main trial as well as in the subanalysis, primarily Caucasian men were enrolled and women were largely underrepresented, limiting the generalisability and reproducibility of the analysis.

Given the small number of participants enrolled in ERASe, blood samples from the baseline and follow-up visit were only available in 36 patients, which reduces the power of the trial. Therefore, the pilot design should be considered as exploratory and hypothesis generating.

The subanalysis is limited to only routine inflammatory parameters, so expanding mechanistic profiling and including cytokines or pro-resolving mediators might play a role in understanding the inflammatory burden as well. Even though we have a significant correlation for one inflammatory biomarker, the probability of further significant interactions for other inflammatory parameters is unlikely and hence, further analysis of specific biomarkers was not performed and would be beyond the scope of this study.

Although, the main trial revealed statistically significance, a smaller sample size of 36 patients was analysed in this post hoc analysis and is underpowered for multiple-association testing. Although findings revealed significant differences, the probability of false positive/negative results is high within the small sample size and type 1, as well as type 2, error cannot be ruled out; hence, results must be interpreted with caution.

The negative binomial regression models were not adjusted for additional covariates because of baseline imbalances between groups and the limited sample size, as further adjustment could have resulted in model overfitting and unstable estimates. Consequently, residual confounding cannot be excluded and should be considered an important limitation of this subanalysis.

## 6. Conclusions

The ERASe trial provides the first clinical evidence worldwide demonstrating beneficial effects of the SGLT2-I Ertugliflozin on the incidence of ventricular arrhythmic burden in patients with chronic heart failure and reduced ejection fraction. As inflammation plays a crucial role in CHF, this subanalysis identified a higher VA burden in Ertugliflozin-treated patients with elevated hsCRP levels, which might suggest a potential association between ventricular arrhythmic risk and inflammatory activity under SGLT2-I therapy. However, this finding is based only on a single significant interaction analysis in a small sample size, and no significant associations were observed for other inflammatory markers. This supports that the observed findings may be incidental and accordingly, should be considered exploratory as well as hypothesis generating, and results must be interpretated with caution.

## Figures and Tables

**Table 1:** Comparison of inflammatory marker between treatment arms.

Inflammatory Markers	Adjusted Means at Follow-Up	Adjusted Mean Relative Difference	Adjusted Percent Difference	*p*-Value
	Mean ± SEM	Mean ± SEM	Mean ± SEM	
**Leukocytes (G/L)**				
Placebo	7.03 ± 0.27	1.07 ± 0.06	7.0 ± 6.3	0.223
Ertugliflozin	7.52 ± 0.29			
**Neutrophils (G/L)**				
Placebo	4.72 ± 0.25	1.06 ± 0.08	6.0 ± 8.3	0.460
Ertugliflozin	4.99 ± 0.26			
**Lymphocytes (G/L)**				
Placebo	1.44 ± 0.08	1.19 ± 0.09	19.0 ± 10.78	0.028
Ertugliflozin	1.72 ± 0.09			
**hsCRP (mg/L)**				
Placebo	2.81 ± 0.69	1.21 ± 0.38	21.0 ± 45.9	0.558
Ertugliflozin	3.39 ± 0.82			
**IL-6 (pg/mL)**				
Placebo	5.76 ± 0.83	1.10 ± 0.23	10.0 ± 25.0	0.631
Ertugliflozin	6.36 ± 0.97			
**NLR**				
Placebo	3.71 ± 0.30	0.874 ± 0.10	−13.0 ± 8.9	0.256
Ertugliflozin	3.24 ± 0.28			
**PLR**				
Placebo	153 ± 10.80	0.892 ± 0.09	−11.0 ± 7.68	0.247
Ertugliflozin	136 ± 9.14			

Values are reported after adjusting for baseline values of markers. Abbreviations: SEM = standard error of mean; hsCRP = high-sensitive C-reactive protein; IL-6 = interleukin-6 = NLR, neutrophil–lymphocyte ratio; PLR = platelet–lymphocyte ratio.

**Table 2:** Negative binomial regression: association of sustained VT/VF episodes at follow-up with change in inflammatory markers from baseline and interaction effects of Ertugliflozin.

Characteristic	IRR	95% CI	*p*-Value
Leukocytes change	1.33	0.86, 2.13	0.118
Interaction: Ertugliflozin * Leukocytes change	0.56	0.18, 1.61	0.226
Lymphocytes change	2.12	0.35, 13.5	0.339
Interaction: Ertugliflozin * lymphocytes change	0.16	0.00, 7.52	0.254
Neutrophils change	1.43	0.88, 2.50	0.056
Interaction: Ertugliflozin * Neutrophils change	0.90	0.18, 4.56	0.857
hsCRP-log change	1.16	0.52, 2.33	0.623
Interaction: Ertugliflozin * hsCRP-log change	3.58	1.12, 11.40	**0.031**
IL-6-log change	1.39	0.28, 6.81	0.608
Interaction: Ertugliflozin * IL-6-log change	4.71	0.53, 46.9	0.075
NLR change	0.98	0.66, 1.70	0.882
Interaction: Ertugliflozin * NLR change	1.47	0.69, 2.62	0.172
PLR change	1.00	0.98, 1.03	0.487
Interaction: Ertugliflozin * PLR change	1.01	0.98, 1.03	0.452

Abbreviations: CI = confidence interval, IRR = incidence rate ratio, hsCRP = high-sensitive C-reactive protein; IL-6 = interleukin-6 = NLR, neutrophil–lymphocyte ratio; PLR = platelet–lymphocyte ratio, VT = ventricular tachycardia; VF = ventricular fibrillation. * Represents the interaction term between Ertugliflozin and the indicated inflammatory marker change. Bold and underlined values highlight the only statistically significant result identified in this analysis (*p* < 0.05).

## Data Availability

The datasets used and/or analysed during the current study is available from the corresponding author on reasonable request.

## Supplementary Materials

The following supporting information can be downloaded at: https://www.mdpi.com/article/10.3390/jcm15124681/s1, Table S1: Baseline characteristics of the post-hoc analysis (n=36); File S1: CONSORT checklist.
