# Supplementary material for: Inflammatory Biomarkers and Their Associations with Arrhythmic Burden Following SGLT2-I Treatment in Chronic Heart Failure—A Subanalysis of the ERASe Trial
Source: J Clin Med. 2026 Jun 17;15(12):4681. doi: 10.3390/jcm15124681 (PMC13302555; doi:10.3390/jcm15124681)
Supplement: Supplementary file 1 [file jcm-15-04681-s001.zip › jcm-4328388-supplementary.pdf]

Table S1. Baseline characteristics of the post-hoc analysis (n=36)

Characteristic	Overall N = 36[1]	Placebo N = 18[1]	Ertugliflozin N = 18[1]	P-value[2]
**Demographic data**				
Age (years)	64 (58, 75)	69 (55, 75)	64 (55,75)	0.195
Sex	33 (92%)	17 (94%)	16 (89%)	>0.999
Systolic blood pressure (mmHg)	137 (127, 147)	135 (126, 146)	137 (130, 149)	0.392
Diastolic blood pressure (mmHg)	84 (75, 97)	78 (68, 91)	88 (82, 98)	0.106
Heart rate (bpm)	67 (59, 77)	70 (59, 83)	66 (58, 74)	0.296
Weight (kg)	88 (78, 97)	89 (78, 95)	85 (79, 99)	0.692
Height (cm)	173 (170, 179)	174 (170, 180)	173 (170, 176)	0.515
BMI (kg/m^2)	27 (25, 32)	27 (23, 31)	27 (26, 33)	0.319
Smoking history	24 (67%)	12 (67%)	12 (67%)	>0.999
Active smoker	4 (19%)	4 (40%)	0 (0%)	0.035
Diabetes	4 (11%)	2 (11%)	2 (11%)	>0.999
Hypertension	25 (69%)	13 (72%)	12 (67%)	0.717
**Baseline medication**				
ACE-I/AT1-RA	20 (56%)	9 (50%)	11 (61%)	0.502
Beta Blocker	30 (83%)	16 (89%)	14 (78%)	0.658
MRA	20 (56%)	10 (56%)	10 (56%)	>0.999
ARNI	10 (28%)	6 (33%)	4 (22%)	0.457
Loop Diuretics	19 (53%)	12 (s67%)	7 (39%)	0.095
Dihydropyridine-CA	4 (11%)	2 (11%)	2 (11%)	>0.999
Non- Dihydropyridine-CA (Verapamil or Diltiazem)	0 (0%)	0 (0%)	0 (0%)	--
Digitalis	1 (2.8%)	1 (5.6%)	0 (0%)	>0.999
Amiodaron	7 (19%)	3 (17%)	4 (22%)	>0.999
PAI	8 (22%)	4 (22%)	4 (22%)	>0.999
Anticoagulation	20 (56%)	9 (50%)	11 (61%)	0.502
Statin	22 (61%)	10 (56%)	12 (67%)	0.494
**Laboratory measurements**				

Characteristic	Overall<br>N = 36[1]	Placebo<br>N = 18[1]	Ertugliflozin<br>N = 18[1]	P-value[2]
**Demographic data**				
Hemoglobin (g/dL)	17 (15, 139)	16 (15, 137)	17 (15, 147)	0.580
HbA1c (mmol/mol)	40.0 (38.0, 42.5)	39.5 (38.0, 41.0)	40.5 (38.0, 46.0)	0.399
eGFR (ml/min/1.73m2)	60 (50, 78)	61 (48, 69)	60 (53, 88)	0.496
Leukocytes (G/L)	7.35 (6.32, 8.71)	7.58 (5.54, 8.88)	7.27 (6.40, 8.60)	0.825
Lymphocytes (G/L)	1.64 (1.27, 2.05)	1.40 (1.20, 1.92)	1.80 (1.30, 2.20)	0.326
Neutrophils (G/L)	4.90 (3.70, 6.10)	5.20 (3.70, 6.40)	4.90 (3.70, 5.70)	0.692
Platelets (G/L)	209 (170, 235)	172 (155, 207)	227 (210, 245)	0.001
NLR	2.73 (2.06, 4.29)	3.12 (2.18, 5.00)	2.56 (2.00, 3.26)	0.367
hsCRP (mg/L)	2.00 (0.90, 3.95)	1.85 (0.90, 3.50)	2.00 (1.00, 5.10)	0.704
PLR	121 (95, 146)	108 (82, 162)	130 (111, 146)	0.126
IL-6 (pg/mL)	4.65 (3.25, 6.70)	5.30 (3.90, 6.90)	4.25 (3.10, 5.90)	0.229
NT-proBNP (ng(L)	551 (241, 2562)	1686 (280, 3340)	363 (175, 1305)	0.048
Sodium (mmol/L)	139 (137, 141)	139.5 (138, 142)	138 (137, 140)	0.212
Potassium (mmol/L)	4.2 (4.1, 4.6)	4.3 (4.1, 4.6)	4.2 (4.0, 4.4)	0.382
Magnesium (mmol/L)	0.83 (0.79, 0.88)	0.83 (0.79, 0.87)	0.85 (0.8, 0.88)	0.620
**Baseline arrhythmic burden**				
Persistent VT/VF	1 (0, 5)	1 (0, 3)	2 (0,8)	0.423
Non-persistent VT/VF	10.5 (4.5, 15)	11.5 (4, 15)	10 (5, 19)	0.610
LVEF (%)	34.75 (28, 43)	33.8 (27, 44)	35 (30, 43)	0.874

[1] n (%); Median (Q1, Q3)

[2] Chi-squared test or Fisher's exact test for categorical variables. Wilcoxon rank sum test for continuous variables

Abbreviations: BMI = body mass index; ACE-I = angiotensin converting enzyme inhibitor; AT1-RA = angiotensin 1breceptor antagonist; MRA = mineralocorticoid receptor antagonist; ARNI = angiotensin receptor neprilysin inhibitor; CA = Calcium antagonist; PAI = platelet aggregation inhibitor; HbA1c = hemoglobin A1c; eGFR = estimated glomerular filtration rate; NLR = neutrophil-lymphocyte ratio; hsCRP = high-sensitive C-reactive protein; PLR = platelet lymphocyte ratio; IL-6 = interleukin 6; NTproBNP = N-terminal pro brain natriuretic peptide; VT = ventricular tachycardia; VF = ventricular fibrillation; LVEF = left ventricular ejection fraction
